# Evaluating the potential for iodinated radiocontrast agents to interfere with ADAMTS13 activity testing via fluorescence resonance energy transfer methodology

**DOI:** 10.1093/ajcp/aqae160

**Published:** 2024-12-05

**Authors:** Jeremy W Jacobs, Melissa S Stuart, Julie I Tange, Rachel R Leger, Aneel A Ashrani, Dong Chen, Rajiv K Pruthi, Meera Sridharan, Jansen N Seheult

**Affiliations:** Department of Pathology, Microbiology & Immunology, Vanderbilt University Medical Center, Nashville, TN, US; Special Coagulation Laboratory, Division of Hematopathology, Department of Laboratory Medicine and Pathology, Mayo Clinic, Rochester, MN, US; Special Coagulation Laboratory, Division of Hematopathology, Department of Laboratory Medicine and Pathology, Mayo Clinic, Rochester, MN, US; Special Coagulation Laboratory, Division of Hematopathology, Department of Laboratory Medicine and Pathology, Mayo Clinic, Rochester, MN, US; Special Coagulation Laboratory, Division of Hematopathology, Department of Laboratory Medicine and Pathology, Mayo Clinic, Rochester, MN, US; Special Coagulation Laboratory, Division of Hematopathology, Department of Laboratory Medicine and Pathology, Mayo Clinic, Rochester, MN, US; Division of Hematology, Department of Internal Medicine, Mayo Clinic, Rochester, MN, US; Department of Pathology, Microbiology & Immunology, Vanderbilt University Medical Center, Nashville, TN, US; Special Coagulation Laboratory, Division of Hematopathology, Department of Laboratory Medicine and Pathology, Mayo Clinic, Rochester, MN, US; Division of Hematology, Department of Internal Medicine, Mayo Clinic, Rochester, MN, US; Special Coagulation Laboratory, Division of Hematopathology, Department of Laboratory Medicine and Pathology, Mayo Clinic, Rochester, MN, US; Division of Hematology, Department of Internal Medicine, Mayo Clinic, Rochester, MN, US; Special Coagulation Laboratory, Division of Hematopathology, Department of Laboratory Medicine and Pathology, Mayo Clinic, Rochester, MN, US

**Keywords:** thrombotic thrombocytopenic purpura, laboratory interference, iodinated contrast media, coagulation testing, ADAMTS13 activity, preanalytical variable, analytic variable, TTP

## Abstract

**Objectives:**

Fluorescence resonance energy transfer (FRET)–based ADAMTS13 activity assays are critical for the diagnosis of thrombotic thrombocytopenic purpura. However, these assays are susceptible to interference. As iodide has been suggested to interfere in laboratory testing via fluorophore quenching or promotion, we aimed to determine whether iodinated contrast (Omnipaque) interferes with the ATS-13 ADAMTS13 Activity Assay 2.0.

**Methods:**

We evaluated the excitation, emission, and absorbance spectrum of Omnipaque alone and spiked in patient plasma with known ADAMTS13 activity and ADAMTS13 activity on Omnipaque alone, an abnormal control of patient plasma previously observed to display elevated baseline relative fluorescent units, and variable concentrations of patient plasma with known ADAMTS13 activity spiked with Omnipaque.

**Results:**

No atypical fluorescent peaks were observed on any sample (Omnipaque alone or spiked in plasma) between 250 and 700 nm. There was no difference in the mean ADAMTS13 activity among the various concentrations of plasma spiked with Omnipaque or plasma spiked with saline.

**Conclusions:**

Iodinated contrast does not appear to interfere—either via spectral overlap of the fluorophore or through physiologic inhibition of the ADAMTS13 enzyme—with ADAMTS13 activity FRET-based assays based on the findings from this in vitro analysis. Delaying sample collection for ADAMTS13 activity testing from suspected patients with thrombotic thrombocytopenic purpura following administration of iodinated radiocontrast agents is not necessary, and recent contrast administration should not yield erroneous ADAMTS13 activity results.

**Key Points**

- Fluorescence resonance energy transfer (FRET)–based assays for ADAMTS13 activity are susceptible to interference from various physiologic and exogenous sources.
- Iodinated contrast (eg, Omnipaque) does not appear to interfere with ATS-13 ADAMTS13 Activity Assay 2.0 using FRET technology, including at supraphysiologic concentrations or after light exposure.
- ADAMTS13 activity testing for patients with possible thrombotic thrombocytopenic purpura using this FRET-based assay should not be delayed due to iodinated contrast administration.

## INTRODUCTION

Thrombotic thrombocytopenic purpura (TTP) is a rare thrombotic microangiopathy characterized by a severe deficiency of ADAMTS13 (a disintegrin and metalloprotease with thrombospondin type 1 repeats, member 13).^1,2^ ADAMTS13 is responsible for cleaving prothrombotic ultra-large von Willebrand factor (ULVWF) multimers; the absence of this enzyme results in the accumulation of these ULVWF multimers and subsequently leads to thrombi formation in the microvasculature.^2-7^ Thrombotic thrombocytopenic purpura occurs most frequently as an immune-mediated condition (iTTP) wherein autoantibodies directed against the ADAMTS13 enzyme inhibit its proteolytic function or induce its accelerated clearance from plasma.^2,8-11^ In contrast to iTTP, congenital TTP accounts for a minority of TTP cases and occurs when mutations in the ADAMTS13 gene cause reduced enzymatic activity (<10%) secondary to altered protein production and/or function.^2,12-14^

Irrespective of the etiology, ADAMTS13 activity less than 10% is considered highly specific for diagnosing TTP (97%-100%, depending on the study and assay methodology).^15-27^ Less severe deficiencies in ADAMTS13 activity may occur in a myriad of conditions, including hepatic dysfunction, severe infection/sepsis, and disseminated intravascular coagulation via mechanisms such as reduced protein synthesis, cleavage of ADAMTS13 by thrombin or plasmin, or quenching of ADAMTS13 activity by bilirubin or hemoglobin.^28-30^ ADAMTS13 activity, therefore, not only is critical to the definitive diagnosis of TTP but also represents a useful test for differentiating TTP from other conditions. ADAMTS13 activity monitoring is also an important component of care for patients in remission, as the results harbor significant therapeutic implications, including dictating when preemptive therapy is employed to prevent clinical relapse.^2,3,15,31-34^

Given that ADAMTS13 activity testing is critical in the diagnostic evaluation and continued management of TTP, accurate results are essential for clinical decision-making. Multiple assays exist for measuring ADAMTS13 activity; most are based on the detection of von Willebrand factor (vWF) cleavage products via enzyme-linked immunosorbent assays (ELISAs) or fluorescence resonance energy transfer (FRET)–based assays incorporating a recombinant vWF substrate.^35-37^ Some of the most common ELISAs incorporate an immobilized glutathione S-transferase–tagged vWF fragment containing the ADAMTS13 cleavage site.^36^ When patient plasma is added, any ADAMTS13 present cleaves the vWF fragment. Subsequent steps include washing, addition of a labeled antibody specific to the cleavage site, and addition of a substrate for the antibody.^36^ A color change is proportional to the ADAMTS13 activity, which is determined by measuring the absorbance at a particular wavelength.^36^ Similarly, there are multiple FRET assays that involve a vWF fragment with a donor fluorophore positioned on one side of the ADAMTS13 vWF cleavage site and a quencher fluorophore located on the other side of the ADAMTS13 vWF cleavage site.^17,36,38^ Cleavage of the vWF fragment by ADAMTS13 in patient plasma uncouples the two fluorophores, allowing unquenched fluorescence by the donor fluorophore, which is proportional to the ADAMTS13 activity.^17^ Understanding the limitations of these assays is paramount to ensuring that reliable results are obtained—for example, free hemoglobin, bilirubin, and/or lipids can interfere with various ADAMTS13 activity assays that use a fluorescent or turbidimetric end point.^39-41^ It has also been shown that antibodies against fluorescent proteins can interfere in ELISA-based assays.^38^ Further, certain endogenous substances, such as bilirubin or neutrophil peptides, may directly inhibit ADAMTS13 activity in vivo and/or in vitro, potentially resulting in falsely decreased results.^42,43^

Another theoretical source of interference is radiocontrast media. These agents are administered to enhance the visibility of internal structures and are used for x-ray–based or magnetic resonance imaging (MRI)–based studies. In the case of x-ray contrast, agents are generally either barium based (typically orally administered) or iodinated (intravenous), while gadolinium-based agents are used in MRI and can be administered orally or intravenously.^44^ Barium sulfate is primarily used for gastrointestinal imaging, while a number of imaging techniques use iodinated contrast (eg, contrast computed tomography [CT], projectional radiography, fluoroscopy).^44^ Iodinated contrast media can be either ionic or nonionic—nonionic contrast media have a lower osmolarity and do not dissociate into charged particles in solution.^45^ This property results in a better safety profile, and therefore these agents are more widely used in contemporary practice.^45^ For example, more than 80 million CT scans are performed in the United States annually, and it is estimated that greater than 40% of these use iodinated contrast, with Omnipaque (iohexol) being the most commonly used contrast medium.^46-48^

Our laboratory, which serves both a large hospital patient population and functions as a major national and international reference laboratory, uses the ATS-13 ADAMTS13 Activity Assay 2.0 (Immucor) for ADAMTS13 activity, a FRET-based assay. We have recently encountered multiple samples with high background fluorescence, resulting in a falsely low ADAMTS13 activity level. We have personally observed that this laboratory effect may potentially be time dependent, with relative fluorescence unit (RFU) elevations increasing upon repeat testing. A chart review of these patients identified iodinated contrast (specifically Omnipaque) as a potential unifying explanation. Notably, iodide has previously been identified as a potential interference in various laboratory assays, including fluorescent-based assays,^49-53^ as it may act as a quencher of some fluorophores and a promoter of other fluorophores.^50^ Therefore, we sought to determine whether this commonly used iodinated radiocontrast media may interfere with ADAMTS13 activity testing using FRET methodology.

## MATERIALS AND METHODS

### Objective

The objective of this study was to determine if a commonly used iodinated contrast agent interferes with ADAMTS13 activity testing by FRET methodology. We selected Omnipaque 300 mg/mL (Omnipaque; GE HealthCare Technologies) as our iodinated contrast agent given that it was administered within 48 hours to all patients that we identified to have falsely decreased ADAMTS13 test results presumably secondary to interference in our retrospective chart review.

Omnipaque 300 mg/mL is an iodinated radiocontrast agent that contains 647 mg iohexol, equivalent to 300 mg of organic iodine per milliliter.^54,55^ Omnipaque is a colorless solution. In normal concentrations, it does not cause a detectable color change to plasma. We evaluated the effect of Omnipaque on ADAMTS13 activity as measured via the ATS-13 ADAMTS13 Activity Assay 2.0 (Immucor). This assay uses a synthetic 73-amino-acid fragment of the vWF A2 domain, and the excitation and emission wavelengths of the fluorophore are 492 nm and 518 nm, respectively.^56^ This study aimed to assess the possibility of both immediate and time-dependent interference of Omnipaque to account for any breakdown products secondary to light exposure, as might occur in the typical laboratory setting when patient samples are left exposed to the ambient environment.

### Fluorescent Spectroscopy

**FIGURE 1:**
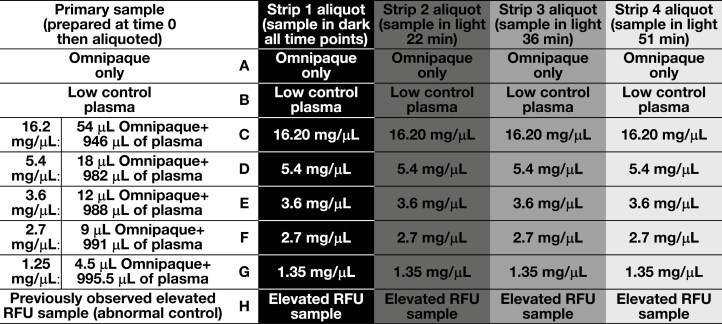
Setup of the plate containing the samples analyzed and the time exposed to light.

Fluorescent spectral analysis was performed to assess the excitation, emission, and absorbance spectra of Omnipaque using an Agilent BioTek Synergy Neo2 Reader (Agilent Technologies). The scan was performed in 2 read sets for excitation and emission. We designed our excitation and emission spectral scan parameters based on the excitation and emission wavelengths of the fluorophore in the ADAMTS13 assay. Thus, the excitation analysis used a fixed emission of 520 ± 15 nm and assessed the excitation spectra every 10 nm from 250 to 490 nm (first read) and 550 to 700 nm (second read). The emission analysis used a fixed excitation of 492 ± 15 nm and assessed the emission every 20 nm from 320 to 465 nm (first read) and 525 to 700 nm (second read). The absorbance spectral analysis was read every 10 nm from 300 to 700 nm. Test wells used 100 mcL of sample in a Nunc FluoroNunc PolySorp 96-well plate (Thermo Fisher Scientific). Samples were maintained in dark and varied-length light environments and then read at approximately 15- to 20-minute intervals. Spectral scans were performed on (1) patient plasma with a known ADAMTS13 activity level (ie, low control), (2) Omnipaque alone, (3) an abnormal control of patient plasma previously observed to display elevated baseline RFUs (ie, abnormal control), and (4) variable concentrations of low control patient plasma with known ADAMTS13 activity spiked with Omnipaque. An Omnipaque concentration was calculated to approximate the expected peak plasma concentration immediately following intravenous (IV) administration of Omnipaque 300 mg/mL for a 70-kg patient undergoing abdominal CT. Three additional plasma concentrations of Omnipaque lower than the peak concentration and 1 Omnipaque concentration 3 times the maximum plasma concentration to represent theoretical IV contamination during specimen collection were also performed. A sample with the same concentration of saline in plasma as the highest concentration of Omnipaque was also used to control for any theoretical dilution of ADAMTS13. FIGURE 1 depicts the setup of the test plate.

### Omnipaque Concentrations

The expected peak plasma concentration for a 70-kg individual receiving a 200-mL maximum dose of Omnipaque 300 mg/mL was calculated to be 5.10 mg/mL (11.0 mg/mL iohexol) based on its volume of distribution (165 mg/mL).^54,55^ We chose to use approximate concentrations to ensure even dilutions with plasma.

The following Omnipaque concentrations were predicted to be observed in patient plasma for the aforementioned hypothetical 70-kg patient undergoing abdominal CT:

- 5.40 mg/mL = 18 mcL Omnipaque in 982 mcL of plasma
- 3.60 mg/mL = 12 mcL Omnipaque in 988 mcL of plasma
- 2.70 mg/mL = 9 mcL Omnipaque in 991 mcL of plasma
- 1.35 mg/mL = 4.5 mcL Omnipaque in 995.5 mcL of plasma

A plasma sample representing hypothetical contamination of the sample with Omnipaque, such as might occur if collected immediately following administration of radiocontrast, was also used:

- 16.20 mg/mL = 54 mcL Omnipaque in 946 mcL of plasma

### ADAMTS13 Activity Testing

Aliquots were made from the above samples and immediately frozen at –70°C. The samples were then thawed for 5 minutes in a 37°C water bath, and ADAMTS13 activity testing was performed with the ATS-13 ADAMTS13 Activity Assay 2.0 (Immucor). Testing was performed on the Janus G3 Workstation (Revvity). The excitation and emission wavelengths of the fluorophore used in this assay are 492 nm and 518 nm, respectively.^56^ We used an excitation of 492 nm and an emission of 520 nm with a 10-nm and 15-nm bandpass, respectively. Samples were tested in duplicate, and the mean activity was reported.

### Statistical Analysis

The mean ADAMTS13 activity of the low control plasma samples spiked with Omnipaque or saline was compared among each other using 1-way analysis of variance with multiple comparisons. The mean ADAMTS13 activity of each of the low control plasma samples spiked with Omnipaque or saline was also compared to the mean ADAMTS13 activity of the low control plasma. An α value of less than .05 was considered statistically significant. All statistical analyses were performed with Prism version 10.2.3 (GraphPad Software).

## RESULTS

**FIGURE 2:**
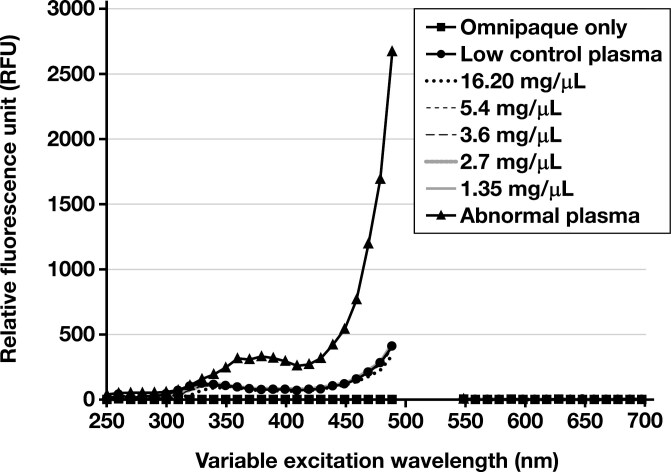
Relative fluorescence unit spectral scan variable excitation spectrum from 250 to 700 nm, with fixed emission at 520 nm. A1 (Omnipaque only), B1 (low control plasma only), C1-G1 (Omnipaque in low control plasma at various concentrations), and H1 (abnormal control plasma sample).

**FIGURE 3:**
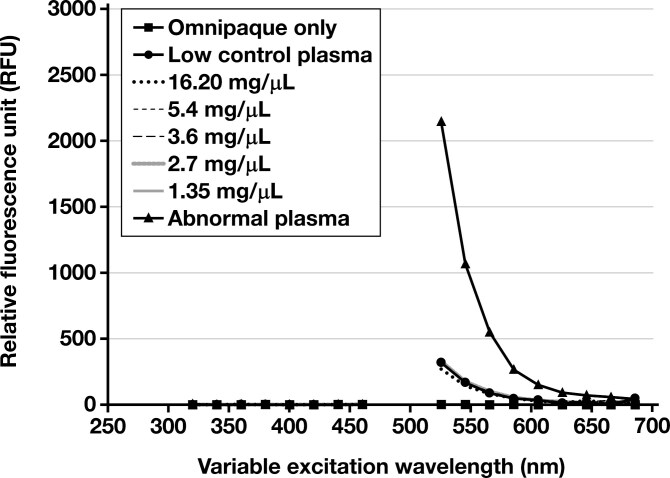
Relative fluorescence unit spectral scan variable emission spectrum from 250 to 700 nm, with fixed excitation at 492 nm. A1 (Omnipaque only), B1 (low control plasma only), C1-G1 (Omnipaque in low control plasma at various concentrations), and H1 (abnormal control plasma sample).

**FIGURE 4:**
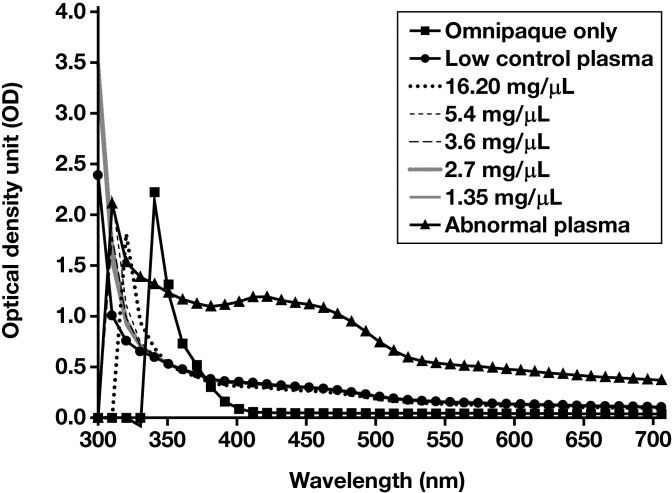
Absorbance spectral scan from 300 to 700 nm. A1 (Omnipaque only), B1 (low control plasma only), C1-G1 (Omnipaque in low control plasma at various concentrations), and H1 (abnormal control plasma sample).

Spectral analysis was performed on samples maintained in the dark (0 minutes) and exposed to light for increasing amounts of time (approximately every 15-20 minutes: 22 minutes, 36 minutes, and 51 minutes). Compared to the abnormal control sample, the excitation and emission spectra demonstrated no fluorescent peaks for the Omnipaque-only sample (A1, FIGURE 2 and FIGURE 3). No atypical fluorescent peaks were observed for the low control plasma only (B1, FIGURE 2 and FIGURE 3) or the low control plasma samples spiked with Omnipaque (C1-G1, FIGURE 2 and FIGURE 3) compared to the known abnormal control patient sample (H1, FIGURE 1 and FIGURE 2). Absorbance spectral scan additionally demonstrated the abnormal control sample to be elevated (H1, FIGURE 4) compared to the Omnipaque only (A1, FIGURE 4), low control plasma only (B1, FIGURE 4), and Omnipaque-spiked low control plasma samples (C1-G1, FIGURE 4).

**FIGURE 5:**
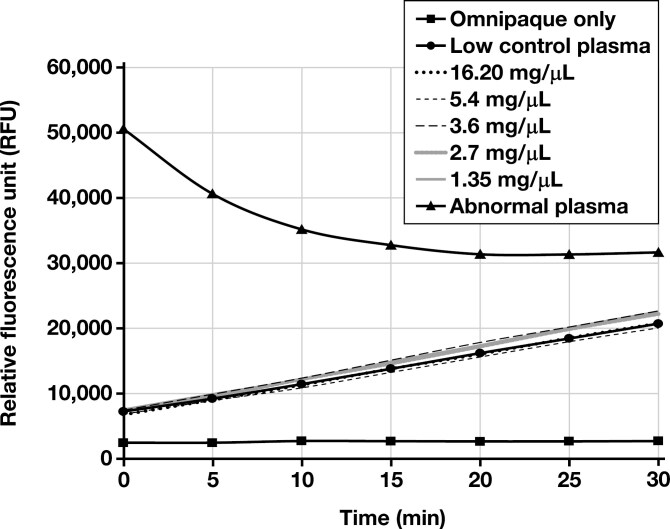
Reaction curves for ADAMTS13 activity testing for the abnormal patient control, low control plasma samples spiked with Omnipaque, and Omnipaque only. The abnormal patient control demonstrated elevated relative fluorescence unit (RFU) at time zero (reaction initiation), which decreased over the course of the reaction. Conversely, the low control patient samples spiked with Omnipaque demonstrated normal reaction kinetics (initial low RFUs that progressed to a higher RFU at the reaction end point). The sample with Omnipaque only demonstrated no significant change in RFUs. All samples were tested in duplicate; the curve that is plotted is the mean of the 2 results for each sample.

In the ADAMTS13 activity testing, none of the spiked plasma samples demonstrated an elevated RFU at T0 (reaction initiation), and all RFUs progressively increased until T30 (reaction termination), demonstrating normal reaction kinetics. These results were congruent with the low control patient sample. Conversely, the abnormal control patient sample demonstrated abnormal reaction kinetics (RFUs higher at T0 than T30) FIGURE 5, TABLE 1.

**TABLE 1:** ADAMTS13 Activity Results^a^

Sample ID	T0 RFU	T30 RFU	Delta RFU	% ADAMTS13 activity	Mean ADAMTS13 activity, %	CV ADAMTS13 activity, %
Omnipaque only	2,479 2,445	2,690 2,725	211 280	0.6 0.7	0.6	10.8
Plasma only (low control)	7,099 7,384	20,175 21,262	13,076 13,878	20.1 21.4	20.8	4.5
16.20 mg/mL Omnipaque	7,045 6,446	21,836 19,708	14,791 13,262	22.9 20.4	21.7	8.2
5.40 mg/mL Omnipaque	6,788 6,869	19,685 20,444	12,897 13,575	19.8 20.9	20.4	3.8
3.60 mg/mL Omnipaque	7,447 7,431	22,325 23,015	14,878 15,584	23.1 24.3	23.7	3.5
2.70 mg/mL Omnipaque	7,345 7,509	21,574 22,822	14,229 15,313	22 23.8	22.9	5.6
1.35 mg/mL Omnipaque	7,446 7,602	22,171 22,925	14,725 15,323	22.8 23.8	23.3	3
Known effect plasma with elevated RFU (abnormal control)	51,872 49,287	31,541 31,746	–20,331 –17,541	<0.0 <0.0	<0.0	UTA
Low control plasma + saline	6,915 7,215	19,726 21,782	12,811 14,567	19.7 22.6	21.1	9.6

CV, coefficient of variation; RFU, relative fluorescence unit; T0, time zero (reaction start time); T30, time 30 (reaction end time); UTA, unable to assess.

^a^The ADAMTS13 activity of the ADM 08 sample was unable to be obtained as the delta RFUs were negative.

**FIGURE 6:**
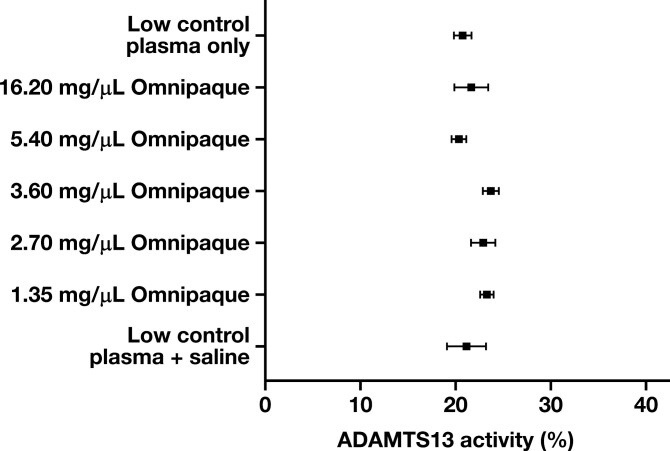
Mean and standard deviation of ADAMTS13 activity in low control plasma only and low control plasma samples spiked with various concentrations of Omnipaque or saline.

There was no difference in the mean ADAMTS13 activity among the various concentrations of low control plasma spiked with Omnipaque or low control plasma spiked with saline TABLE 1, FIGURE 6. When compared with the low control plasma sample, the mean ADAMTS13 activity in the low control plasma samples spiked with Omnipaque was higher at all Omnipaque concentrations, except at 5.40 mg/mL (low control plasma ADAMTS13 activity 0.4% higher than the spiked sample) TABLE 2. This difference was not statistically significant. The only spiked sample with a statistically significant difference in ADAMTS13 activity compared to the low control plasma was at an Omnipaque concentration of 3.60 mg/mL (2.95% greater ADAMTS13 activity in the spiked sample compared to the low control plasma) TABLE 2. This difference was not clinically significant as it approximated the typical assay variation (1 SD ± 2%).

**TABLE 2:** Comparison of Mean ADAMTS13 Activity Difference From the Low Control Plasma (Plasma Only) and Each Test Sample of plasma-Spiked Omipaque or Saline Blank

Sample	Difference in ADAMTS13 activity (low control plasma compared to sample)	95% CI of difference	*P* value
0.0 mg/mL Low control plasma + saline	–0.40	–19.8 to 19.0	.97
1.35 mg/mL Omnipaque	–2.55	–6.19 to 1.09	.07
2.70 mg/mL Omnipaque	–2.15	–8.22 to 3.92	.14
3.60 mg/mL Omnipaque	–2.95	–4.16 to –1.74	.02
5.40 mg/mL Omnipaque	0.40	–2.03 to 2.83	.29
16.20 mg/mL Omnipaque	–0.90	–47.0 to 45.2	.98

## DISCUSSION

This study found that Omnipaque did not alter the ADAMTS13 activity in any of the spiked plasma samples by a clinically significant amount, and only 1 sample (Omnipaque concentration of 5.40 mg/mL) showed an ADAMTS13 activity lower (–0.4% difference) than that of the low control plasma. Reaction RFUs for all Omnipaque-spiked plasma samples demonstrated normal kinetics compared to the known abnormal control sample. Additionally, Omnipaque did not absorb or emit fluorescence within the tested range, paralleling findings from prior studies showing that iodine has an excitation peak at approximately 203 to 214 nm,^51,57^ well below the excitation (492 nm) and emission (518 nm) wavelength of the fluorophore used in the ATS-13 ADAMTS13 Activity Assay 2.0 tested herein. Moreover, based on the reported excitation (340 nm) and emission (450 nm) wavelength of the fluorophore used in the first-generation ATS-13 ADAMTS13 activity assay,^58^ it is unlikely that iodine (and Omnipaque) would produce spectral interference in this assay as well. Additionally, these results are the first to demonstrate that time-dependent exposure of iodinated contrast to an ambient laboratory environment does not result in fluorescent absorbance or emission, including from any theoretical degradation products.

This study did not elucidate the cause of interference in the ADAMTS13 activity assay observed in a subset of patients at our institution; when we encounter this phenomenon, we release an interpretative report stating the inability to provide a definitively accurate ADAMTS13 activity due to interference. Nevertheless, these findings illustrate that physiologic and supra-physiologic levels of iodinated contrast, as encountered after administration for radiologic imaging, are exceedingly unlikely to interfere with FRET-based ADAMTS13 activity assays. This is notable, as the acute presentation of TTP may evoke suspicion of various localized or systemic conditions necessitating imaging evaluation, such as CT of the head, chest, and/or abdomen, and many of these studies use iodinated contrast media.

There are limitations to this study, as in vitro analyses may not entirely recapitulate in vivo processes. However, Omnipaque displays a low affinity for serum or plasma proteins and is poorly bound to serum albumin, and no significant metabolism, deiodination, or biotransformation occurs.^54^ Furthermore, we attempted to reproduce the expected physiologic plasma concentrations of Omnipaque soon after contrast administration, as peak plasma iodine levels are expected to occur immediately following IV injection and fall rapidly within the first hour.^54^ Omnipaque distributes in the extracellular compartment, and approximately 90% of the injected dose is excreted unchanged by glomerular filtration within the first 24 hours. Thus, our spiking studies are expected to represent the typical amount and concentration of Omnipaque in a patient within the first hour up to 24 hours following administration. Additionally, we tested plasma samples that were kept in a dark environment and then subsequently exposed to light over a 1-hour time period to account for any theoretical light-induced degradation or interference from breakdown products, given that we have previously observed a time-dependent interference in patient samples. Similarly, we tested samples that had been frozen and thawed to account for any perturbations secondary to the freeze-thaw process. Finally, although we only tested Omnipaque, and there is no evidence to suggest that similar iodinated contrast formulations would behave differently, we acknowledge that other agents exist, and we cannot definitively conclude they would not display interference in the ADAMTS13 activity assay.

In summary, these results highlight the lack of apparent interference—either via spectral overlap of the fluorophore or through physiologic inhibition of the enzymatic activity of the ADAMTS13 protein—by iodinated radiocontrast media in ADAMTS13 activity FRET assays. We believe these results are likely generalizable to most, if not all, FRET-based ADAMTS13 assays. Thus, delaying sample collection for ADAMTS13 activity testing from suspected patients with TTP following administration of iodinated radiocontrast agents is not necessary, and recent contrast administration should not yield erroneous ADAMTS13 activity results.
